# 

**Published:** 1868-11

**Authors:** 


### CONTENTS.

ORIGINAL COMMUNICATIONS.

The Life of the Trichina.......... 443
Microscopy ot the Teeth.......... 461
Filling Teeth.......... 466
Rose Pearl.......... 470
Dental Laws.......... 472

SELECTIONS.

The Goodyear Rubber Cases.......... 476

EDITORIAL.

"A Single Experiment".......... 485
Our Table of Societies.......... 485
Wales' Objectives.......... 486
Webster's Unabridged.......... 486

